# Field and Experimental Investigations on the Effect of Reservoir Drill-In Fluids on Penetration Rate and Drilling Cost in Horizontal Wells

**DOI:** 10.3390/gels9070510

**Published:** 2023-06-24

**Authors:** Neamat Jameel, Jagar A. Ali

**Affiliations:** 1 Department of Natural Resources Engineering and Management, School of Science and Engineering, University of Kurdistan Hawler, Erbil 44001, Iraq; nimet.jamil@gmail.com; 2 Department of Petroleum Engineering, Faculty of Engineering, Soran University, Soran P.O. Box 624, Iraq; 3 Department of Geology, Palacký University, 17. Listopadu 12, 77146 Olomouc, Czech Republic

**Keywords:** reservoir drill-in fluid, FLOPRO, non-damaging fluid, salt polymer mud, fluid loss, rate of penetration, drilling cost

## Abstract

In this study, the reservoir drill-in fluid (RDF) was modified and optimized to improve the rheological properties and reduce the filtration properties of the drilling fluid used for drilling the oil-bearing zone horizontally. In polymer science, degradation generally refers to a complex process, by which a polymeric material exposed to the environment and workload loses its original properties. Degradation is usually an unwanted process. In certain cases, however, controlled polymer degradation is useful. For instance, it can improve the processability of the polymer or can be used in recycling or natural decomposition of waste polymer. Thus, the drilling fluid and parameter data of 30 horizontal wells that were drilled in the south of Iraq were collected using several reservoir drill-in fluids (RDFs), including FLOPRO, salt polymer mud (SPM), non-damaged fluid (NDF), and FLOPRO_PTS-200 (including the polymer thermal stabilizer). The obtained results showed that the polymer temperature stabilizer (PTS-200) enabled reducing the filtration rate by 44.33% and improved the rheological properties by 19.31% as compared with FLOPRO. Additionally, the average cost of NDF and SPM drilling fluids for drilling the horizontal section of the selected wells is around USD 96,000 and USD 91,000, respectively. However, FLOPRO-based drilling fluid showed less cost for drilling the horizontal section, which is USD 45,000.

## 1. Introduction

Reservoir drill-in fluids (RDFs) made from biopolymers and synthetic polymers have a variety of applications, such as fluid loss reduction, viscosity improvement, and suspension stability [1,2,3]. Bio- and synthetic polymers deteriorate at high-temperature ranges within the harsh conditions of the wellbore [4,5,6,7]. On the other hand, while drilling with water-based mud (WBM), certain polymers are frequently employed to reduce filtration loss in permeable intervals. Although they frequently operate well, they are unable to completely limit the fluid loss and mud invasion into the reservoir, and they may seriously damage the formation, including negative changes in surface wettability [8,9]. Biopolymers are used to make up reservoir drilling fluid, such as non-damaging fluid (NDF) and salt polymer mud (SPM). Therefore, during drilling under High-Pressure High-Temperature (HPHT) conditions, they are not suitable, as the polymer breaks down at the high bottom hole temperature of the well. Hence, a continuous treatment is needed, for the polymer breaks down, which increases the cost of the drilling [10,11,12,13]. However, FLOPRO can be used to overcome the high-temperature problem of drilling inside the borehole [14].

Developments in science and technology, especially over the last two decades, have led to the production of several synthetic polymers worldwide that have resistance to higher temperatures, such as FLOTROL polymers that are used to make up FLOPRO drilling fluid. FLOTROL polymers are more stable at higher temperatures, with less chemical concentration, non-formation damage, zero non-productive time for drilling fluid, and the ability to drill more than 2000 m horizontally at a cost that is significantly lower than commercial polymers [15,16].

The FLOPRO fluid is a proven water-based drill-in system with non-damaging characteristics, a compatibility breaker, an extremely low friction factor for low pump pressures, high ROP, and extremely high capacity for cuttings transport, and a global environmental compliance application revealed that this fluid had consistent performance and rheology. It was suited for drilling horizontal wells because of its good hole cleaning, preventing the development of cutting beds [17,18]. The main components of the FLOPRO solution are fresh (or sea) water, calcium carbonate, the polymer preparations FLO-VIZ and FLO-TROL, sodium or potassium salts, and LUB lubricating additives. FLOPRO is used to provide the lowest skin damage of productive horizons with deviated and horizontal wells. Predicting formation damage in cased-hole and open-hole completion wells is important since the primary objective of reservoir drill-in fluids (RDFs) is to minimize formation damage and provide a thin filter cake that can be removed by differential pressure. This is especially important when the damage is well-bore induced and is brought on by reservoir drill-in fluids. Cake filter removal has been shown to be an effective method for determining induced damage and gauging the effectiveness of drill-in fluids [19,20].

Table 1 illustrates the summary of the performed studies on the application of different types of polymers in the drilling fluid circulation. Samavati and Abdullah (2015) discovered that using gilsonite at concentration of 17.5 gm/cm^3^ reduced the polymer breakdown by 72%, improving the viscosity and decreasing the fluid loss compared with the starch under HPHT conditions [17]. In 2019, Aruther and co-workers stated that adding a novel high-temperature polymer within WBM at a concentration of 7 ppb can increase the thermal stability of WBM to be able to withstand 400 °F and maintain its original properties [21]. In addition, two biopolymers, A and B, were reported to positively influence the fluid loss and formation damage reduction by 60% compared with the clay-free starch-containing drilling mud [8]. Moreover, Akpan et al. (2018) investigated the effect of polyglycol on the drilling fluid rheological properties at the concentration of 0.7 wt.%, and their results showed that the polyethylene glycol additive maintained the suspension capability of the drilling fluid formulations. These additives can be used to stabilize the water-based drilling fluids containing biopolymers at 150–232 °C without using expensive and formation-damaging synthetic polymers [14]. Recently, Wallace and co-workers (2020) studied the effect of the polymeric rheology modifier at the concentration of 5 mg in 350 mL with polymer thermal stabilizer at 2 vol.%. Their outcome shows that synthetic polymer holds more excellent rheology suspension than xanthan gum by 48% [22]. The primary objective of this study is to optimize the types and concentrations of chemicals/additives used to develop reservoir-friendly drilling fluids with high tolerance to temperature to horizontally drill specific sections of the Mishrif and Saadi formations that are in the Basra province in the south of Iraq. For this purpose, the field data and laboratory measurements of several RDFs used in the horizontal interval of 30 wells in the south of Iraq were analyzed. Several drilling parameters, including the lost circulation, buildup volume, drilling cost, rate of penetration, and drilling fluid rheological and filtrations properties, were considered.

## 2. Results and Discussion

### 2.1. Rheological Properties

In this section, the rheological measurements—including the plastic viscosity (µp), apparent viscosity (µa), yield point, and gel strength of the FLOPRO; non-damaging fluid (NDF); salt polymer mud; and FLOPRO with PTS-200 drilling fluids—are presented and discussed. The measurements included plastic viscosity, yield point, and gel strengths at 10 s and 10 min under different temperature conditions, which are shown in Table 2. As can be seen, the plastic and apparent viscosities of the base sample are 13 and 7 cP, respectively, while the yield point is 25 and 14 lb/100 ft^2^, and the 10 s and 10 min gel strengths were 8 and 12 lb/100 ft^2^, respectively.

**Figure 1:**
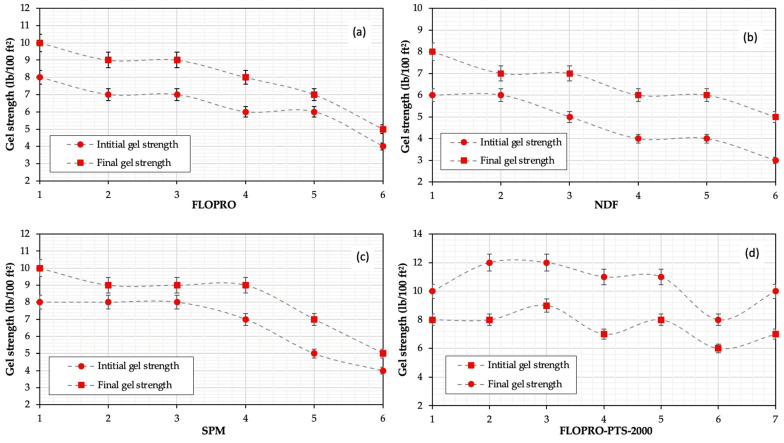
The results of the gel strength at 10 s and 10 min of the reservoir drilling fluid: (**a**) FLOPRO, (**b**) non-damaging fluid, (**c**) salt polymer mud, and (**d**) FLOPRO with PTS-200.

**Figure 2:**
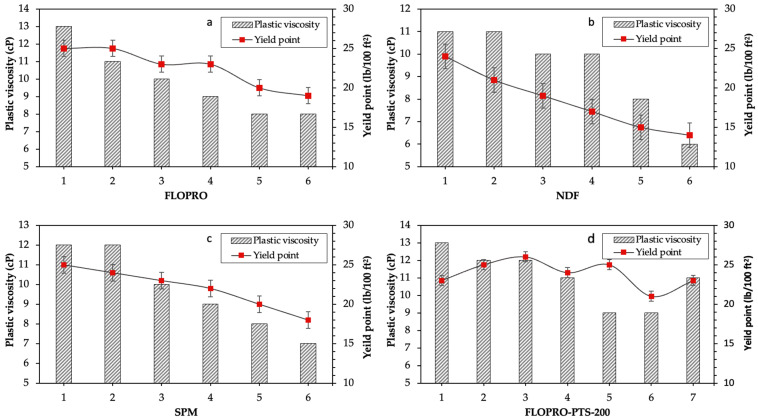
The results of the Yield Point of the reservoir drilling fluid (RDF): (**a**) FLOPRO, (**b**) non-damaging fluid, (**c**) salt polymer mud, and (**d**) FLOPRO with PTS-200.

The rheological properties of 6 prepared samples of FLOPRO drilling fluid with 1.4 lb/bbl of FLO-VIS powder, 6 samples of salt polymer mud drilling fluid with 1.4 lb/bbl of DUO-VIS powder, 6 samples of non-damaging fluid prepared with 1.6 lb/bbl of DUO-VIS powder, and 7 samples of FLOPRO with different concentrations of PTS-200 are shown in Table 2 and Figure 1 and Figure 2. In the sample of FLOPRO drilling fluid, the plastic viscosity (PV) started from 13 cP and then reduced to 8 cP. However, the gel strengths at 10 s and 10 min were also found to be 8 lb/100 ft^2^, but after the temperature increased, the gel strength was reduced to 4 lb/100 ft^2^, and the gel strength at 10 min started from 10 lb/100 ft^2^ reduced to 5 lb/100 ft^2^ due to polymer breaking down. Furthermore, the yield point was also reduced in the sample of FLOPRO drilling fluid starting from 25 lb/100 ft^2^ reduced to 19 lb/100 ft^2^ (see Figure 1a and Figure 2a). In the sample of non-damaging fluid (RDF), the temperature effect on rheological properties, including the plastic viscosity (PV), started from 11 cP and then reduced to 6 cP, which means losses of 45.45% on their specific with temperature formed are fragile for this mud. Nevertheless, the gel strength of 10 s started at 6 lb/100 ft^2^ and then reduced to 3 lb/100 ft^2^, and the gel at 10 min started at 8 lb/100 ft^2^ and then reduced to 5 lb/100 ft^2^.

In addition, the yield point also reduced from 24 to 14 lb/100 ft^2^, which means losses of 41.66% on their rheological properties with a high-temperature shape are frail for this mud (see Figure 1b and Figure 2b). In the sample of salt polymer mud RDF, the plastic viscosity was found from 12 lb/100 ft^2^, then reduced to 7 lb/100 ft^2^. However, the gel strength was affected by the temperature: gel 10 s from 8 lb/100 ft^2^ reduced to 4 lb/100 ft^2^ and gel 10 min from 10 to 5 lb/100 ft^2^. Furthermore, the yield point was reduced from 25 to 18 lb/100 ft^2^ (see Figure 1c and Figure 2c). Polymer temperature stabilizer (PTS-200) was employed to protect the polymer from breaking down and added to the FLOPRO sample; for plastic viscosity, the result shows that it was precisely 20.08% more stable than FLOPRO without PTS-200, and salt polymer and NDF, about 26.28 and 30.07%, respectively. Furthermore, the gel strength of both 10 s and 10 min FLOPRO with PTS-200 is more stable when compared with FLOPRO without PTS-200, NDF, and salt polymer mud about 49.7, 44, and 37.5% separately. Moreover, the yield point of FLOPRO with PTS-200 also showed better performance than FLOPRO without PTS-200, NDF, and salt polymer mud about 24, 41.66, and 28, respectively (see Figure 1d and Figure 2d).

### 2.2. Filtration Properties and Filter Cake Thickness

For the filtration characteristics to obtain better filtration control, 2 types of polymers added to each RDF of the FLOPRO drilling fluid (RDF) were prepared with 6.3 lb/bbl of FLOTROL and 3.5 lb/bbl of M-I PAC UL powder. In total, 6 samples were prepared: salt polymer mud drilling fluid (RDF) prepared with 5 lb/bbl of M-I PAC UL and 4.2 lb/bbl of Polysal powder was used for six samples; Non-Damaging Fluid (RDF) was prepared with 6.8 lb/bbl of PAC LV and 5 lb/bbl of Starch powder and also used for 6 samples. The materials were studied and determined under different temperature ranges from 220 to 500 °F. Table 3 displays the HP/HT fluid losses of three reservoir drilling fluids. As is obvious, the fluid loss is increasing with the increasing of the temperate from 220 to 500 °F.

**Figure 3:**
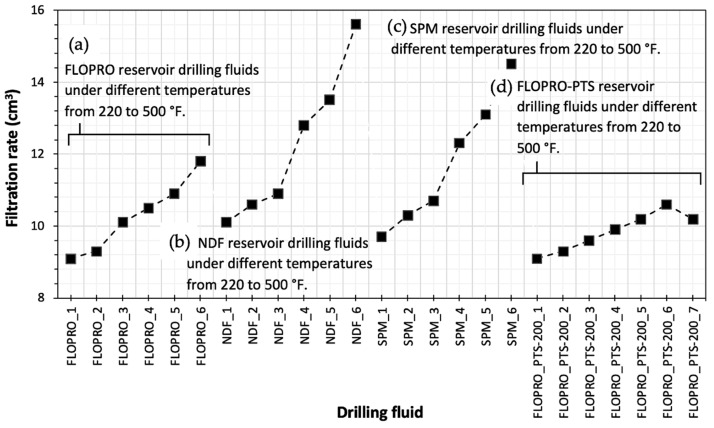
Measured filtration rates at 30 min of the developed reservoir drilling fluids (RDFs) under different temperatures from 220 to 500 °F: (**a**) FLOPRO, (**b**) non-damaging fluid, (**c**) salt polymer mud, and (**d**) FLOPRO with PTS-200.

Figure 3 illustrates the filtration rate of the FLOPRO, non-damaging fluid, salt polymer mud, and FLOPRO with PTS-200 drilling fluid for 30 min. As can be seen, the NDF fluid had the highest filtration rate, which increases with temperature to 15.6 mL at 30 min. Generally, the salt polymer mud drilling fluids prepared from M-I PAC UL and Polysal powders showed better performance in reducing the filtration rate due to creating sufficient filter cakes, which are impermeable at about 14.5 m. While the FLOPRO drilling fluids were prepared with FLOTROL and M-I PAC, fluid losses of 11.8 mL were recorded. In addition, PTS-200 after being added to FLOPRO showed the best performance; the minimum filtration rate of 10.2 mL at 30 min was obtained. During the investigation of the drilling fluid with the optimum temperature for polymer before breaking down, it was found that FLOTROL started to break down at 320 °F; meanwhile, drilling was required to treat the active system. However, PAC LV and M-I PAC UL polymer started to break down at 280 °F. Furthermore, Polysal polymer started to break down at 170 °F. Moreover, adding 2 wt.% of PTS-200 to FLOPRO (RDF) at 500 °F obtained the best performance to reduce filtration and protect the polymer from breaking down when the previous sample of FLOPRO at 500 °F with 1.5% PTS-200 was about 10.78% (see Figure 3).

**Figure 4:**
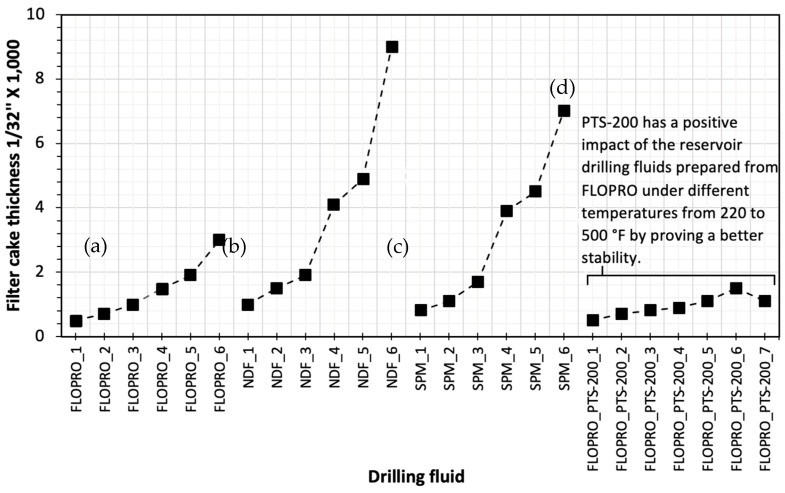
Measured filter cake thicknesses of 3 types of reservoir drilling fluids (RDFs) under different temperatures from 220 to 500 °F: (**a**) FLOPRO, (**b**) non-damaging fluid, (**c**) salt polymer mud, and (**d**) FLOPRO with PTS-200.

Furthermore, Figure 4 illustrates the filter cake thickness of three types of the reservoir drilling fluids; FLOTROL filtration polymer shows better resistance to temperature, and the thickness increased by 83%. However, in the sample of non-damaging fluid, polysal showed poor results, with the lowest resistance to the temperature and a higher filtration rate at 500 °F. Moreover, the thickness of the filter cake increased by 94.44%. Furthermore, the sample of salt polymer mud showed slightly better resistance to the temperature, and the filter cake thickness was thinner than non-damaging fluid, but thicker than FLOPRO; the result showed 92.85%. Moreover, adding a polymer temperature stabilizer (PTS-200) to RDF can protect the polymer from breaking down by 66.66%, but the filter cake thickness increases by 40%.

### 2.3. Drilling Parameters

#### 2.3.1. Rate of Penetration

**Figure 5:**
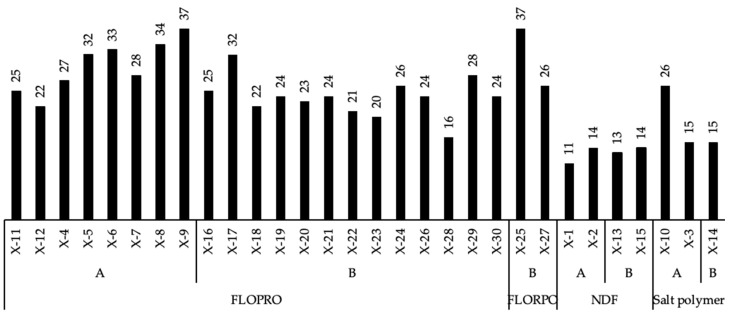
Average rate of penetration (ROP) of drilling the horizontal section of the reservoir of Wells X-1 to 30 using FLOPRO, non-damaging fluid, and salt polymer reservoir drilling fluids.

Overall, FLOPRO drilling fluid was used to drill 22 wells, whereas salt polymer mud (SPM) was used to drill 4 wells and non-damaging drilling fluid to drill 4 wells. The well was drilled using FLOPRO with a maximum ROP of 37 m/h for Well X-9. The minimum ROP was 16 m per hour in Well X-16. The well’s highest ROP while using NDF was 14 m per hour in Well X-14, and the minimum ROP was 11 m/h in Well X-1. Furthermore, the greatest ROP for the salt polymer-drilled well was 26 m/h. The minimum ROP was 15 m per hour, as shown in Figure 5. Figure 5 displays the ROP for all wells that were drilled utilizing the three different types of RDF. Among all the used drilling fluids, the maximum ROP of 37 m/h was obtained when FLOPRO was used in drilling Wells X-8 and X-25. However, NDF illustrated the minimum ROP of 11 m/h in Well X-11. Overall, NDF reduced the ROP in all four wells that were used for drilling the horizontal section of the reservoir.

#### 2.3.2. Cost per Meter (USD/m)

**Figure 6:**
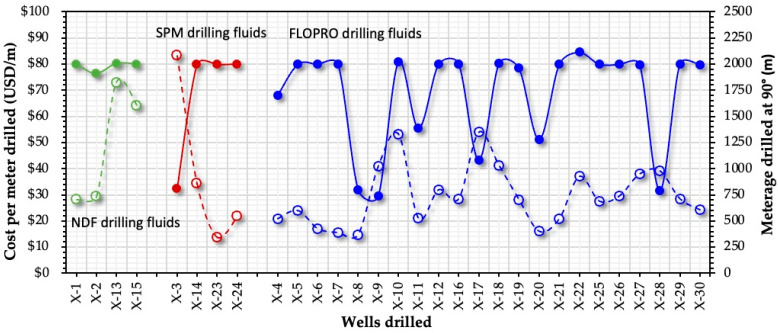
Measured depth and the cost per meter (USD/m) of drilling the horizontal section of the reservoir of Wells X-1 to 30 using FLOPRO, non-damaging fluid, and salt polymer reservoir drilling fluids.

The cost per meter calculation of the drilling fluid used for drilling the horizontal section of thirty wells, excluding the drilling rig and other services in drilling, is shown in Figure 6. FLOPRO’s average cost is roughly USD 29.64, compared with salt polymer mud’s average cost of USD 38.28 and NDF’s average cost of USD 48.67. As a result, FLOPRO is more affordable and performs better compared with the other types of used reservoir drilling fluids. The overall cost of the FLOPRO drilling fluid supplied for drilling about 2000 is 41.52% less than the SPM drilling fluid supplied for 4 wells and the NDF drilling fluid used in 4 wells. The highest cost of USD 83.3 for drilling a meter of the reservoir section horizontally was recorded for Well X-14 using SPM drilling fluid when only 811 m were drilled. However, the minimum cost of USD 14.8 for drilling a meter of the reservoir horizontally was obtained when drilling Well X-8 using FLOPRO reservoir drilling fluid when drilling 798 m (see Figure 6). In addition, the number of meters drilled of the reservoir section horizontally are shown in Figure 6. The horizontal section in almost all wells is about 2000 m. The minimum number of meters were drilled in Well X-9, which is 736 m, and the maximum was 2114.5 m in Well X-22.

#### 2.3.3. Formation Losses

**Figure 7:**
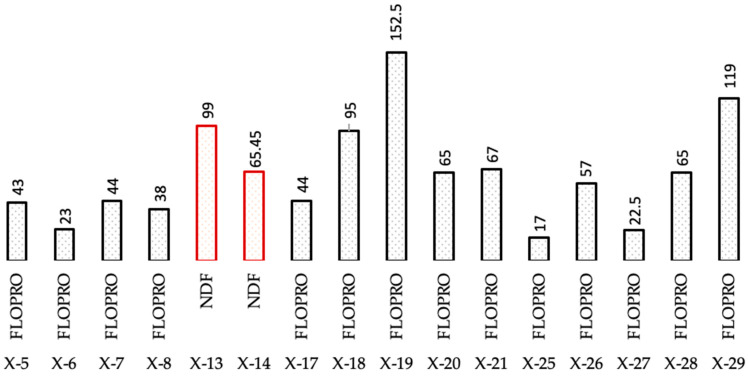
Total losses of the used drilling fluid in the reservoir section of studied wells.

Overall, 30 wells were drilled with 3 different types of reservoir drilling fluids. Drilling with salt polymer mud resulted in no downhole losses, whereas drilling with NDF resulted in 2 wells with average losses of 82.22 m^3^. In addition, 14 FLOPRO-drilled wells experienced downhole losses, with an average losses rate of roughly 60.85 m^3^, as shown in Figure 7. As is clear, the highest losses of 152.2 m^3^ happened within Well X-19, which was drilled using FLOPRO drilling fluid, while Well X-25 showed a minimum loss of the FLOPRO drilling fluid of 17 m^3^. The loss of NDF drilling fluid is also high in both Wells X-13 and X-14, which was 99 and 65.45, respectively.

### 2.4. Cost Analysis

#### 2.4.1. Cost of Reservoir Section, Volume Build-Up, and Cost Per Barrel (USD/bbl)

**Figure 8:**
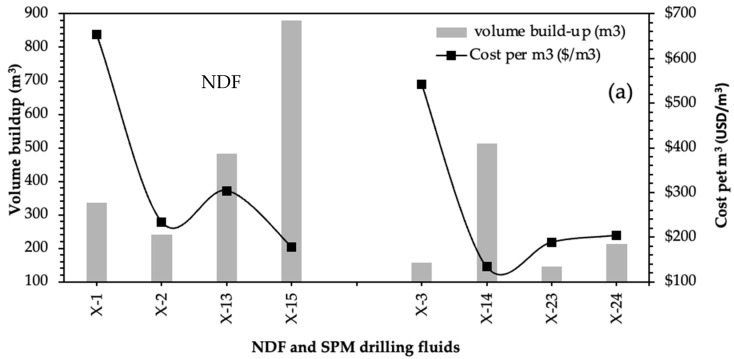

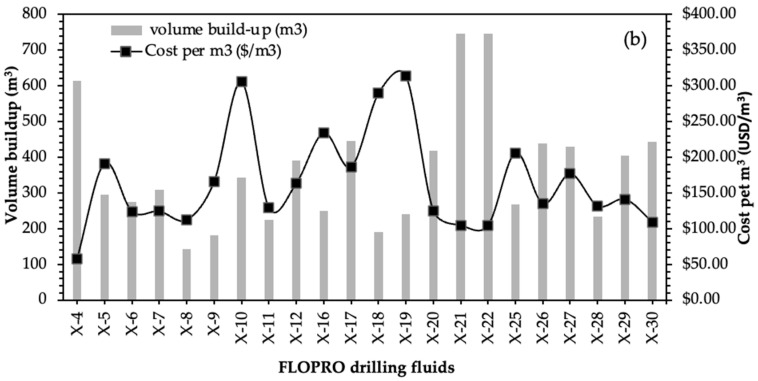
Volume buildup and its cost of the used reservoir drilling fluids in 30 wells: (**a**) SPM and NDF drilling fluids and (**b**) FLOPRO drilling fluid.

NDF drilling fluid was used in Wells X-1, 2, 13, and 15, and SPM RDF was used in Wells X-3, 14, 23, and 24, as shown in Figure 8a, while FLOPRO drilling fluid was used in 22 wells (see Figure 8b). The average costs of NDF and SPM drilling fluids were 342 and 267 USD/m^3^, respectively, and USD 165.11 was the average cost of each cubic meter of FLOPRO drilling fluid used in drilling the reservoir section horizontally. Hence, FLOPRO’s end-of-well cost is significantly lower than that of NDF and salt polymer. The volume buildup of NDF and SPM drilling fluids was starting from 146.5 m^3^ used in Well X-23 to the highest amount of 880 m^3^ in Well X-15, as shown in Figure 8a. Furthermore, the costs of each cubic meter of SPM and NDF RDFs used in Well X-14 and Well X-1 are 133.57 and 653 USD/m^3^, respectively. The FLOPRO buildup volume varied from 143 to 445.5 m^3^, along with its cost per m3, which started from 58 to 314 USD/m^3^ (see Figure 8b).

#### 2.4.2. Completion Cost (USD/bbl)

**Figure 9:**
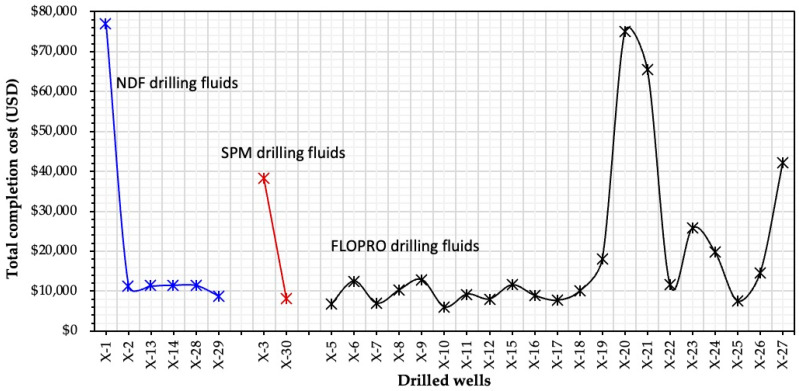
Measured values of the completion cost of 30 wells with types of reservoir drilling fluid.

Wells that were drilled with FLOPRO fluid had a lower overall completion cost compared with those drilled with NDF and SPM fluids because the NDF needed a D-Destroyer system to remove the filter cake from the borehole wall, and SPM needed a CaCl_2_ filtration unit to complete the Saadi Formation. The NDF fluid was used in the completion of Wells X-1, 2, 13, 14, 28, and 29, and the maximum cost was recorded for Well X-1, which is USD 76,844. The cost of other wells by NDF was much less, that is, around USD 10,000. In addition, the completion cost of almost all wells by FLOPRO is around USD 10,000, expect for Wells X-22 and X-23. The average completion cost with FLOPRO is USD 8034 for all 20 wells used, from the minimum of USD 9945 to the maximum rate of USD 75,072. Hence, the average cost of the completion with FLOPRO is lower compared with the NDF and SPM fluids, which are USD 21,818 and USD 16,947, respectively (see Figure 9).

### 2.5. Challenges and Prospective the Reservoir Drill-in Fluid

The breakdown temperature of polymers depends on the type of polymer and the drilling conditions. Some polymers, such as starch, can break down at temperatures as low as 150 degrees Fahrenheit. Others, such as FLOTROL, can withstand temperatures up to 300 degrees Fahrenheit when a polymer temperature stabilizer (PTS-200) is added. PTS-200 is a solution that can benefit modern industry challenges and promote the strategic opportunities of oil and gas companies [28,29,30,31,32].

It is important to take steps to protect polymers from breaking down. One of the main challenges in the oil industry is the lifecycle of polymers in long-term and short-term projects. As long as polymers break down, continuous treatment is required to keep drilling fluid parameters in the acceptable range. At the end, the well concentration and cost will be much higher than the planned cost of drilling the well [33,34,35,36,37,38].

After the end of a recent project, data were collected and compared from 30 turnkey project wells that used 3 different types of drilling fluid (RDF). The results indicated that FLOPRO had a positive economic impact and improved the KPI performance for drilling parameters. However, the study was limited by the fact that no core samples were taken during the drilling operation. This means that there was no core sample that could be used in the lab to test the filter cake removal performance of FLOTROL. Additionally, PTS-200 was not used in the NDF and salt polymer mud.

Overall, the results of this study suggest that FLOPRO is a promising new RDF that can help to improve drilling efficiency and reduce its costs. However, further research is needed to confirm the filter cake removal performance of FLOTROL and to assess its impact on skin damage.

## 3. Conclusions

The main goal of this study was to formulate a drilling fluid using an optimum concentration of polymer temperature stabilizer (PTS-200) to protect the polymers from breaking down at a temperature greater than 220 degrees Fahrenheit in order to achieve better filtration and rheological properties and lower cost. During the investigation period, starch polymer, an anti-filter loss polymer, began to degrade at 170 °F, whereas FLOTROL (modified starch) degraded at 320 °F. The optimum concentrations of polymer temperature stabilizer (PTS-200) were discovered at a temperature above the 320 degrees Fahrenheit required for 1% of PTS-200, based on achieving the lowest filtration rate. However, wells with greater temperatures, such as 500 °F, required using 2 wt.% of PTS-200, based on the archiving of better filtration and rheological properties. The results showed that polymers used to make FLOPRO, including FLOTROL and M-I PAC UL, are more stable than those used to make NDF and salt polymer mud, such as starch and PAC LV, which will break down at temperatures over 320 °F and result in viscosity that is 13.66% less stable and 31.14% less suitable for filtering.

## 4. Materials and Methods

### 4.1. Materials

Various additives were used in this study, such as modified natural (Duo-Vis) and synthetic viscosifier (FLO-VIS), sodium carbonate (Na_2_CO_3_), starch, biopolymer filtration (Polysal), poly anionic cellulose ultra-low viscosity (M-I PAC UL), polyanionic cellulose low-viscosity (PAC LV), synthetic polyanionic cellulose low-viscosity (FLOTROL) polymer, caustic soda (NaOH), NaCl salt, chloromethyl-isothiazolinone (M-I Cide), low-cost surfactant, drilling torque reducer (drillzone), ester lubricant (Lube XLS) and water-soluble brine lubricant (Safe Lube), phosphate-base corrosion inhibitor (Qonqor 404), and polymer temperature stabilizer (PTS-200). Each of the above chemicals and additives was added for a specific purpose, such as fluid loss control, viscosity improvement, corrosion inhibition, lubricity improvement, and temperature stability. All the mentioned chemicals were provided by the M-I SWACO—Schlumberger company (Basra, Iraq) with a purity of 99.8%.

### 4.2. Field Data and Study Area

Field data of drilling horizontal sections of 30 wells (X-1 to X-30) in the south of Iraq were collected. The drilled reservoir sections were Mishrif Formation from the middle cretaceous and Saadi Formation from the late cretaceous, which are limestone white chalky. The thicknesses of the Saadi and Mishrif formations are 136 m (from 2017 to 2153 m MD) and 110 m (from 2242 to 2352 m MD), respectively. Both formations are drilled horizontally at a 90° angle with an open-hole section length of about 2000 m. Table 4 shows the collected data from wells X-1-30. As can be seen, two drilling rigs, A and B, were used for drilling these wells. In addition, the meterage drilled, true of vertical depth (TVD), times taken to drill the sections, and horizontally drilled meterage are shown.

### 4.3. Preparation of the Drilling Fluids

Excluding the base sample, four types of RDFs were prepared. The developed RDFs are categorized into FLOPRO (six samples), salt polymer mud, SPM (six samples), non-damaging fluids, NDF (six samples), and an additional seven samples of FLOPRO with polymer temperature stabilizer (PTS-200). All drilling fluids were prepared using a hot plate stirrer and Hamilton Beach mixer for 30 min. The composition of the formulated drilling fluids is shown in Table 5, which includes the concentration of each chemical/additive used within each different drilling fluid sample.

### 4.4. Rheological Measurements of the Drilling Fluids

An API standard viscometer (FANN 35) was used to measure the rheological parameters of all prepared drilling fluid samples, including apparent viscosity, plastic viscosity, yield point, and gel strength under different temperature conditions of 220 to 500 °F. The apparent viscosity, plastic viscosity, and yield point are calculated using readings of 300 and 600 RPM of the viscometer rotor using Equations (1)–(3). The device is modified to measure gel strengths at 10 s and 10 min by observing the greatest, or maximum, deflection of the dial prior to the gel breaking. The recorded deflections of the rotating viscometer at various speeds allowed for the determination of shear loads, shear rates, and drilling fluid’s 10 s and 10 min gel strengths.

Plastic Viscosity (µp) (cP) = 600 rpm reading − 300 rpm reading(1)

Apparent Viscosity (µa) (cP) = 600 rpm reading/2(2)

Yield Point (τy) (Ib/100 ft2) = 300 rpm reading − µp(3)

### 4.5. Filtration Measurements of the Drilling Fluids

The filtration properties of the prepared drilling fluids at different concentrations of FLOTROL, M-I PAC UL, polysal, starch, and PAC LV were studied using a Series 300 HPHT Filter Press (M-I SWACO—Schlumberger company, Basra, Iraq) at 600 psi and 220–500 °F. The drilling fluid sample was put into the filter cell for each of the samples depicted in Table 5. The filter press is set up for the tests, and a graduated cylinder is positioned beneath the filtrate tube. As soon as the equipment is ready, timing is started, and the test begins. The test may continue for 30 min, and the amount of filtrate in the graduated cylinder was recorded.

### 4.6. Analysis of Drilling Field Data

**Figure 10:**
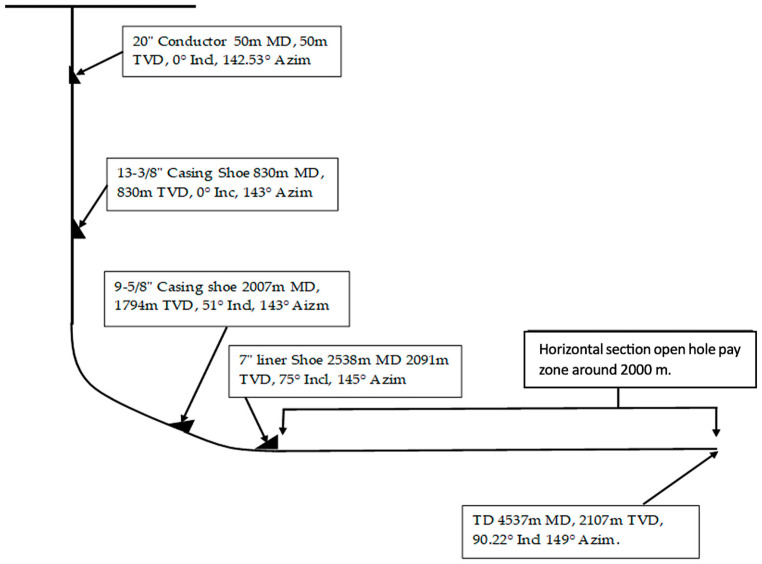
Well profile includes size and depth of casing, and a horizontal section of the reservoir that field data are taken from.

Field data collected from 30 horizontal wells are for all the different types of RDFs mentioned in Table 5; NDF was used in drilling Wells X-1, 2, 13, and 15; SPM was used within Wells X-3, 14, 2,3, and 24; and FLOPRO was used in other wells. The profile of Well X-1 is shown in Figure 10, which includes a horizontal section of the reservoir that the field data are taken from. However, the rest of the wells almost have the same well profile. The mentioned drilling fluids were used under different temperature conditions through the reservoir interval of 2000 m horizontally. Field data will be focusing on the rate of penetration (ROP), cost of drilling in the reservoir section, cost per meter (USD/m), completion cost, volume build-up, cost per barrel (USD/bbl), and downhole losses using the following formulas:

Cost per meter (USD/m) = Cost of reservoir section (USD)/meterage drilled (m) (4)

Rate of Penetration (m/h) = Meterage drilled in reservoir section/day to complete section(5)

Cost per barrel (USD/bbl) = Cost of reservoir section/volume build-up(6)

## Figures and Tables

**Table 1:** Summary of the published research studies investigated on polymer temperature stabilizer, synthetic polymer, and biopolymer within the reservoir drilling fluid.

Reference	Polymer Temperature Stabilizer	Concentration	Mechanism	Study Outcomes
Samavati and Abdullah [17]	Gilsonite	17.5 cm^3^/350 mL	Rheological improvement	Combined with thermal stabilizers, improved the viscosity of WBM by 22% and lowered fluid losses by 25%.
Galindo et al. [18]	Novel high-temperature polymer	7 ppb	Rheological improvement	WBM can withstand temperatures of 400 °F while maintaining its viscosity, excellent suspension, low shear strengths, shale stability, and filtration control by 18%.
Akpan et al. [15]	Polyglycol	0.7 wt.%	Rheological and filtration improvement	Synthetic polymers to stabilize water-based drilling fluids containing biopolymers improved viscosity by 6%, and the filtration rate was reduced by 14%.
Tehrani et al. [23]	PAC	6 gm of PAC in 350 mL	Rheological improvement	The efficiency with 46% fluid loss reduction and the highest value of plastic viscosity, yield point, and gel strength was around 34%.
Al-Otaibi et al. [24]	Xanthan gum and glycol	3 mg in 350 mL	Rheological improvement	Increased the plastic viscosity by 38% at 170–280 °F.
Huang et al. [25]	Laponite	25 and 50 wt.%	Resistance to temperature improvement	Laponite could increase the onset deposition temperature of solid-state AAD terpolymer and substantially increase the high-temperature viscosity of 2 wt.% AAD terpolymer water solution.
Zaboli et al. [19]	Hydrophobic silica NPs	2 wt.%	Resistance to temperature improvement	Hydrophilic or hydrophobic silica NPs phase separation occurred after only a few minutes. By contrast, the emulsions containing the modified silica NPs with contact angles around 92 and 115 were stable for months and days, respectively.
Chen H, et al. [26]	Novel hyper-cross-linked polymer (ACP)	3 gm in 400 mL	Filtration improvement	The preferred one (ACP-5) can reduce the filtrate volume of oil-based drilling fluid by over 90% with a small dosage (3 g in 400 mL drilling fluid) after hot rolling for 16 h at 840 °F.
Zhang et al. [27]	Viscosity stabilizer (PB-854)	2-tert-Butylphenol, paraformaldehyde, phloroglucinol 2:1:2.5	Resistance to temperature improvement	The results show that PB-854 has good high-temperature stability and could effectively protect the polymer at the high temperature.

**Table 2:** Measured values of the plastic viscosity (µp), apparent viscosity (µa), yield point, and gel strength (10 s and 10 min) of the FLOPRO, non-damaging fluid, salt polymer mud, and FLOPRO with PTS-200 drilling fluid.

Drilling Fluid	Sample	Temperature	YP	µ_p_	Gel Strength (lb/100 ft^2^)	
		°F	(Ib/100 ft^2^)	(cP)	Gel_inital_	Gel_final_
FLOPRO	FLOPRO_1	220	25	13	8	10
	FLOPRO_2	280	25	11	7	9
	FLOPRO_3	320	23	10	7	9
	FLOPRO_4	360	23	9	6	8
	FLOPRO_5	400	20	8	6	7
	FLOPRO_6	500	19	8	4	5
NDF	NDF_1	220	24	11	6	8
	NDF_2	280	21	11	6	7
	NDF_3	320	19	10	5	7
	NDF_4	360	17	10	4	6
	NDF_5	400	15	8	4	6
	NDF_6	500	14	6	3	5
SPM	SPM_1	220	25	12	8	10
	SPM_2	280	24	12	8	9
	SPM_3	320	23	10	8	9
	SPM_4	360	22	9	7	9
	SPM_5	400	20	8	5	7
	SPM_6	500	18	7	4	5
FLOPRO with PTS-200	FLOPRO_PTS-200_1	220	23	13	8	10
	FLOPRO_PTS-200_2	280	25	12	8	12
	FLOPRO_PTS-200_3	320	26	12	9	12
	FLOPRO_PTS-200_4	360	24	11	7	11
	FLOPRO_PTS-200_5	400	25	9	8	11
	FLOPRO_PTS-200_6	500	21	9	6	8
	FLOPRO_PTS-200_7	500	23	11	7	10

**Table 3:** Measured values of the HPHT fluid losses with temperature applied, including initial and final thickness of filter paper, and filter cake thickness of the FLOPRO (RDF), non-damaging fluid, salt polymer mud, and FLOPRO with PTS-200 drilling fluid.

Drilling Fluid	Sample	Temp.	HPHT Fluid Loss	Filter Cake Thickness		
				Initial	Final	Average
		°F	cm^3^/30 min	1/32″	1/32″	1/32″
FLOPRO	FLOPRO_1	220	9.1	0.004	0.0045	0.0005
	FLOPRO_2	280	9.3	0.004	0.0047	0.0007
	FLOPRO_3	320	10.1	0.004	0.005	0.001
	FLOPRO_4	360	10.5	0.004	0.0055	0.0015
	FLOPRO_5	400	10.9	0.004	0.0059	0.0019
	FLOPRO_6	500	11.8	0.004	0.007	0.003
NDF	NDF_1	220	10.1	0.004	0.005	0.001
	NDF_2	280	10.6	0.004	0.0055	0.0015
	NDF_3	320	10.9	0.004	0.0059	0.0019
	NDF_4	360	12.8	0.004	0.0081	0.0041
	NDF_5	400	13.5	0.004	0.0089	0.0049
	NDF_6	500	15.6	0.004	0.009	0.005
Salt Polymer mud	SPM_1	220	9.7	0.004	0.0048	0.0008
	SPM_2	280	10.3	0.004	0.0051	0.0011
	SPM_3	320	10.7	0.004	0.0057	0.0017
	SPM_4	360	12.3	0.004	0.0079	0.0039
	SPM_5	400	13.1	0.004	0.0085	0.0045
	SPM_6	500	14.5	0.004	0.011	0.007
FLOPRO with PTS-200	FLOPRO_PTS-200_1	220	9.1	0.004	0.0045	0.0005
	FLOPRO_PTS-200_2	280	9.3	0.004	0.0047	0.0007
	FLOPRO_PTS-200_3	320	9.6	0.004	0.0048	0.0008
	FLOPRO_PTS-200_4	360	9.9	0.004	0.0049	0.0009
	FLOPRO_PTS-200_5	400	10.2	0.004	0.0051	0.0011
	FLOPRO_PTS-200_6	500	10.6	0.004	0.0055	0.0015
	FLOPRO_PTS-200_7	500	10.2	0.004	0.0051	0.0011

**Table 4:** Field data were collected from 30 wells in South Iraq using RDFs through the reservoir horizontally.

No.	Rig	Well	TVD	Meterage Drilled (MD)	Days to Finish Well	Meterage Drilled at 90°
1	A	X-1	2137	4867	56	1997
2	A	X-2	2405.88	4590	60	1910
3	A	X-3	2134	3520	37	811
4	A	X-4	2369.15	4686	38	1700
5	A	X-5	2397.75	4778	26	2000
6	A	X-6	2383.62	4770	27	2000
7	A	X-7	2416.96	5114	27	2000
8	A	X-8	2413.85	3729	22	798
9	A	X-9	2431.47	4176	22	736
10	A	X-10	2387	4826	47	2019
11	A	X-11	2408.23	4114	41	1386
12	A	X-12	2366	4946	46	2000
13	B	X-13	2379	5015	76	2005
14	B	X-14	2364.13	5456	52	1995
15	B	X-15	2477.01	5120	54	2000
16	B	X-16	2371	4690	46	2003
17	B	X-17	2367	3767	24	1081
18	B	X-18	2373.31	4855	25	2005
19	B	X-19	2368.1	4917	23	1960
20	B	X-20	2379.77	4434	35	1276
21	B	X-21	2427.79	4839	30	1997
22	B	X-22	2442.46	5571	45	2114.5
23	B	X-23	2371	4684	39	1997
24	B	X-24	2106.39	4523	31	2000
25	B	X-25	2368	4681	26	2000
26	B	X-26	2370	5026	42	1997
27	B	X-27	2363.5	4695	35	1995
28	B	X-28	2135	3411	37.8	789
29	B	X-29	2371	4690	46	2003
30	B	X-30	2413.54	5201	31	1995

**Table 5:** Composition of the formulated reservoir drill-in fluids used in this study.

Drilling Fluid Component	Reservoir Drilling Fluid (RDF)									
	FLOPRO	SPM	NDF	FLOPRO_PTS-200						
				1	2	3	4	5	6	7
Water, mL	350	350	350	350	350	350	350	350	350	350
Soda ash, gm	0.7	0.7	0.7	0.7	0.7	0.7	0.7	0.7	0.7	0.7
Caustic Soda, gm	0.5	0.5	0.7	0.5	0.5	0.5	0.5	0.5	0.5	0.5
FLO-Vis, gm	1.4	-	-	1.4	1.4	1.4	1.4	1.4	1.4	1.4
FLOTROL, gm	6.3	-	-	6.3	6.3	6.3	6.3	6.3	6.3	6.3
Safe Carb-20, gm	14	-	-	14.0	14.0	14	14	14	14	14
Safe Lube, cm^3^	2.0	1.5	-	2.0	2.0	2.0	2	2	2	2
M-I Cide, cm^3^	0.2	0.2	-	0.2	0.2	0.2	0.2	0.2	0.2	0.2
M-I PAC UL, gm	3.5	-	5.0	3.5	3.5	3.5	3.5	3.5	3.5	3.5
Qonqor 404, cm^3^	1.0	-	-	1.0	1.0	1.0	1.0	1.0	1.0	1.0
DUO-Vis, mg	-	1.6	1.4	-	-	-	-	-	-	-
PAC LV, mg	-	6.8	-	-	-	-	-	-	-	-
CaCO_3_ F, mg	-	14.0	14.0	-	-	-	-	-	-	-
Starch, mg	-	5.0	-	-	-	-	-	-	-	-
NaCl, mg	-	-	1.11	-	-	-	-	-	-	-
Lube XLS	-	-	2.0	-	-	-	-	-	-	-
DrillZone, cm^3^	-	-	1.0	-	-	-	-	-	-	-
Polysal, mg	-	-	4.2	-	-	-	-	-	-	-
ZnCO_3_, mg	-	-	0.7	-	-	-	-	-	-	-
PTS-200	-	-	-	-	0.5	1.0	1.0	1.5	1.5	2.0

## Data Availability

The authors can provide any necessary information on the study upon request.
